# Design and Experimental Study of Longitudinal-Torsional Composite Ultrasonic Internal Grinding Horn

**DOI:** 10.3390/mi14112056

**Published:** 2023-11-02

**Authors:** Hongyin Zhang, Feng Jiao, Ying Niu, Chenglong Li, Ziqiang Zhang, Jinglin Tong

**Affiliations:** School of Mechanical and Power Engineering, Henan Polytechnic University, Jiaozuo 454003, China; 212105010023@home.hpu.edu.cn (H.Z.); niuying@hpu.edu.cn (Y.N.); lichenglong2020@yeah.net (C.L.); 18339183809@163.com (Z.Z.); tongjinglin@hpu.edu.cn (J.T.)

**Keywords:** horn, longitudinal-torsional vibration, finite element analysis, ultrasonic vibration grinding, surface roughness

## Abstract

Longitudinal-torsional composite ultrasonic vibration has been widely used in grinding. This paper aims to solve the problem that the resonance frequency deviates greatly from the theoretical design frequency and the vibration mode is poor when the horn is matched with a larger tool head. This paper presents how the longitudinal-torsional composite ultrasonic conical transition horn was designed and optimized by the transfer matrix theory and finite element simulation. For this purpose, the spiral groove parameters were optimized and selected by finite element simulation. Then, the modal analysis and transient dynamic analysis of the horn with grinding wheel were carried out to verify the correctness of the theoretical calculation. The impedance analysis and amplitude test of the horn with grinding wheel were carried out. The test results were in very good agreement with the theoretical and simulation results. Finally, the grinding experiment was carried out. The surface roughness of the workpiece in longitudinal-torsional ultrasonic vibration grinding was obviously reduced compared to that of ordinary grinding. All these obtained results demonstrate that the designed longitudinal-torsional composite ultrasonic horn has very good operational performance for practical applications.

## 1. Introduction

In recent years, ultrasonic vibration machining technology has been widely used in the aerospace, automotive, shipbuilding, medical and other processing fields [1]. Some difficult-to-machine materials such as ceramics, alloys, and composite materials using ultrasonic processing technology have achieved better processing results than by traditional processing methods [2]. The ultrasonic horn is one of the most critical and important parts in ultrasonic vibrational machining systems. Its function is to amplify the input vibration signal and output it to the tool head, so that the tool head can realize vibration machining [3]. The ultrasonic composite methods mainly include longitudinal-torsional ultrasonic composites [4,5], longitudinal-bending ultrasonic composites [6], bending-torsional ultrasonic composites, and radial-torsional ultrasonic composites [7]. In the field of grinding, ultrasonic vibration-assisted grinding employs an ultrasonic horn connected to the grinding wheel, so that the grinding wheel can realize vibration grinding. In this way, better surface quality can be obtained compared with ordinary grinding [8,9]. Longitudinal-torsional composite vibration is widely used in many ultrasonic machining fields [10,11,12]. In the grinding process, it is beneficial to solve the problem of grinding wheel blockage and grinding burn, reduce the grinding force [13,14], and improve the grinding quality and grinding efficiency [15]. Therefore, it is particularly important to design a set of ultrasonic horns that match the grinding wheel used for actual processing.

At present, the design methods of ultrasonic horn mainly include the following: an analytical method, a substitution method, a transfer matrix method [16,17], and a finite element method [18]. The reasonable design of a longitudinal-torsional composite vibration horn is very important for the practical application of ultrasonic machining. Asami et al. [19] studied a new type of ultrasonic longitudinal-torsional vibration source. It consisted of a longitudinal transducer and a torsional transducer, which were placed at both ends of a uniform rod as a vibration source, to separately control each vibration type, resulting in a composite vibration source located within a planar locus. Deen et al. [20] proposed a longitudinal and torsional vibration actuator for a rotary impact ultrasonic drill (RPUD) for rock sampling in asteroid exploration. Based on finite element modal analysis and harmonic analysis, the sensitivity of the displacement amplitude of the step horn and the driving tips to changes in the structural parameters were analyzed. The resonance frequency and vibration displacement were measured, and the experimental results were in good agreement with the finite element analysis results. Bie et al. [21] discussed the influence of the ultrasonic incident angle on the longitudinal-torsional composite horn. Based on the theory of elastic wave field, the causes of modal transformation and the vibration characteristics of the horn were analyzed. The spiral grooves with different angles were set on the conical section of the horn. The vibration mode of the output end-face of the horn was analyzed by the finite element analysis method, and a drilling test was carried out. The experimental results showed that the larger the longitudinal-torsional ratio of the ultrasonic amplitude, the smaller the average drilling force, and the better the surface roughness of the hole. Zhong et al. [22] proposed a longitudinal-torsional composite consolidation vibration system. The influence of the structural parameters of the composite horn on the frequency of the consolidation vibration system was analyzed, and the structural parameters of the composite horn were optimized. Li et al. [23] designed a longitudinal-torsional ultrasonic horn (HWLTD) with a large tool head, and predicted and optimized the resonant frequency. On this basis, the principle of spiral groove vibration mode conversion was deduced. Combined with the principle of vibration mode transformation and ANSYS simulation analysis, the resonance frequency and the torsional-longitudinal ratio were optimized.

When the traditional method is used to design a horn with larger tool head (such as a large diameter impeller, grinding wheel, etc.), the ultrasonic vibration energy is transferred to a larger load, which will not only lead to a sharp decline in amplitude, but also lead to a large difference between the resonant frequency of the overall vibration and the design frequency [23]. Therefore, this paper combined both the transfer matrix theory and finite element analysis to model the horn with grinding wheel and carry out the finite element simulation optimization design, which is of great significance for improving the vibration characteristics of the horn and the tool head [24,25].

In order to solve the problem of large deviation with theoretical design frequency and poor vibration shape after applying the grinding tool head, this paper first uses the transfer matrix theory and finite element analysis to design and optimize the conical transition longitudinal vibration ultrasonic horn. Next, the structural parameters of the spiral groove are optimized by single factor simulation analysis. Then, the finite element analysis and vibration performance test of the conical transition longitudinal-torsional composite ultrasonic horn are carried out to verify the correctness of the theoretical design and simulation analysis. Finally, a longitudinal-torsional ultrasonic vibration internal grinding experiment using GCr15 steel was carried out. The influence of ultrasonic amplitude on the grinding surface roughness was analyzed, and the processing performance of the horn was verified. The research results of this paper can provide reference for the design of a longitudinal-torsional ultrasonic composite horn with tool head.

## 2. Design of Conical Transition Longitudinal-Torsional Composite Ultrasonic Horn

### 2.1. The Principle of Longitudinal-Torsional Ultrasonic Vibration Internal Grinding

**Figure 1:**
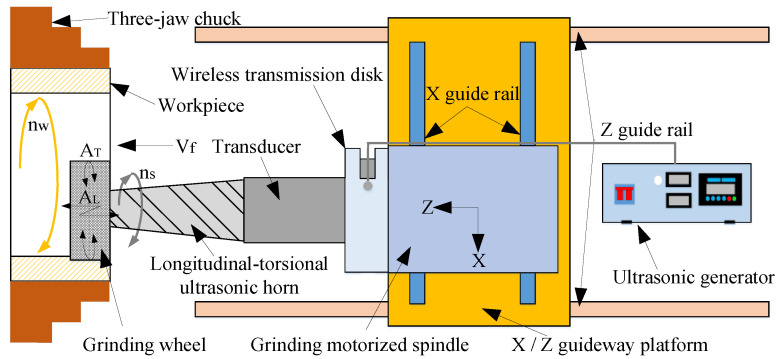
The principle of longitudinal-torsional ultrasonic vibration grinding.

Figure 1 shows a schematic diagram of longitudinal-torsional ultrasonic vibration internal grinding. The processing tool is the grinding wheel, which performs both rotary and longitudinal feed motions. The rotation speed is n_s_ and the longitudinal feed speed is v_f_. The object being processed is a ring workpiece with an inner circular surface, which is rotated at a speed of n_w_. The horn with a spiral groove is used to connect it to the grinding wheel, to which it transmits the longitudinal ultrasonic vibration resulted from the combined action of the ultrasonic generator, wireless transmission disk and transducer. The action of the spiral groove converts the longitudinal ultrasonic vibration into a longitudinal-torsional composite ultrasonic vibration, so that the grinding wheel realizes longitudinal-torsional ultrasonic vibration grinding.

A large number of research and experimental results show that compared with ordinary grinding, longitudinal-torsional ultrasonic vibration-assisted grinding can effectively solve the problems of grinding wheel blockage and grinding burn, reduce grinding force, improve grinding quality and grinding efficiency, and prolong tool life [26,27].

### 2.2. Design and Optimization of the Longitudinal Vibration Ultrasonic Horn

In the selection of horn materials, the characteristics and mechanical properties of materials should be considered comprehensively. Titanium and titanium alloys are widely used in many fields due to their high specific strength, excellent corrosion resistance, low density, and low magnetic susceptibility [28]. They have high strength and excellent comprehensive properties. Therefore, a TC4 titanium alloy was selected as the material for the horn.

In order to realize the longitudinal-torsional ultrasonic composite vibration grinding and obtain the larger amplification coefficient and shape factor of the horn, a conical transition longitudinal-torsional ultrasonic composite horn was selected [29]. When selecting the design frequency of the horn, the influence of the diameter of the grinding tool head, the diameter of the transducer, the length of the horn, the amplification coefficient and the shape factor should be considered.

For the full wavelength horn, the calculation of the wavelength *λ* is as follows:

(1) $$\lambda =\frac{\sqrt{E/\rho }}{f}$$

For the actual processing work in this paper, if the length of the horn is too short, it cannot meet the use requirements, and if too long, will lead to insufficient stiffness. Therefore, we chose a horn frequency range of 25–30 kHz.

For transducer diameter *D*, *D* is generally required to be less than *λ*/4. Then, when *f* = 30 kHz, *λ* = 164.62 mm can be obtained. Due to the influence of the grinding tool head, the diameter of the transducer should be at least greater than 30 mm. Therefore, the range of transducer diameter *D* is:

(2) $$30\;\mathrm{mm}\leq D\leq \frac{\lambda }{4}\;=\;41.16\;\mathrm{mm}$$

Considering the influence of the amplification factor and the shape characteristics of the horn, a design frequency of 28 kHz was selected. Hence, a 28 kHz piezoelectric ceramic transducer with a diameter *D* = 38 mm was employed in our design, while the tool head was a CBN grinding wheel with diameter of 50 mm, width of 15 mm, and made of a titanium alloy matrix.

According to the literature [25,30,31], in this paper, the requirements for vibration and impedance characteristics of the horn are shown in Table 1.

**Figure 2:**
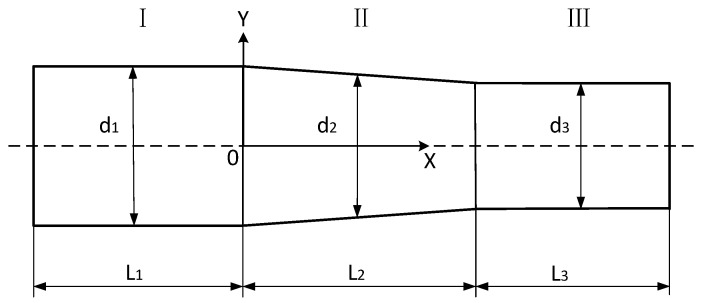
The conical transition of the longitudinal-torsional ultrasonic horn.

Then the design of the conical transition longitudinal-torsional ultrasonic horn is started, and the model is shown in Figure 2.

As shown in Figure 2, the overall structure of the conical transition longitudinal vibration ultrasonic horn is composed of a large cylindrical section I, a conical transition section II and a small cylindrical section III. The lengths and diameters of the three parts of I, II and III are *L*_1_, *L*_2_, *L*_3_ and *d*_1_, *d*_2_, *d*_3_, respectively. The longitudinal vibration excitation is input on the large cylindrical section I, and the whole structure resonates in the one-dimensional longitudinal direction. The force and vibration velocity on all sections are continuous, and the impedance transfer matrix method can be used to calculate the size parameters of the horn [32].

The area function of each section can be expressed as:

(3) S=S(x)

The area function of I and III is expressed as $S_{1}$ and $S_{3}$, respectively. The area function of conical transition section II, $S_{2}\left(x\right)$, can be expressed as:

(4) $$\frac{S_{2}\left(x\right)}{S_{1}}=\frac{\pi {\left[\frac{d_{2}\left(x\right)}{2}\right]}^{2}}{\pi {\left(\frac{d_{1}}{2}\right)}^{2}}={\left[\frac{d_{2}\left(x\right)}{d_{1}}\right]}^{2}$$

(5) $$S_{2}\left(x\right)=S_{1}\cdot {\left[\frac{d_{2}\left(x\right)}{d_{1}}\right]}^{2}$$

From Figure 2, the diameter of the conical transition section II $d_{2}\left(x\right)$ can be expressed as:

(6) $$d_{2}\left(x\right)=d_{1}-\frac{d_{1}-d_{3}}{L_{2}}x$$

Substitute Equation (6) into Equation (5):

(7) $$S_{2}\left(x\right)=S_{1}\cdot {\left(1-\frac{d_{1}-d_{3}}{d_{1}L_{2}}x\right)}^{2}$$

Equation (7) can be simplified as follows:

(8) $$N=\sqrt{\frac{S_{1}}{S_{3}}}=\frac{d_{1}}{d_{3}}$$

(9) $$\alpha =\frac{d_{1}-d_{3}}{d_{1}L_{2}}=\frac{N-1}{NL_{2}}$$

(10) $$S_{2}\left(x\right)=S_{1}\cdot {\left(1-\alpha x\right)}^{2}$$

where α is the taper coefficient of the conical section and N is the area coefficient.

The transfer matrix expression of the longitudinal vibration of the horn is as follows [32]:

(11) $$\left[\begin{array}{c} F_{3}\\ v_{3} \end{array}\right]=\left[\begin{array}{cc} a_{11}^{1} & a_{12}^{1}\\ a_{21}^{1} & a_{22}^{1} \end{array}\right]\left[\begin{array}{cc} a_{11}^{2} & a_{12}^{2}\\ a_{21}^{2} & a_{22}^{2} \end{array}\right]\left[\begin{array}{cc} a_{11}^{3} & a_{12}^{3}\\ a_{21}^{3} & a_{22}^{3} \end{array}\right]\left[\begin{array}{c} F_{1}\\ v_{1} \end{array}\right]=Z_{1}Z_{2}Z_{3}\left[\begin{array}{c} F_{1}\\ v_{1} \end{array}\right]$$

where *Z*_1_, *Z*_2_ and *Z*_3_ are the impedance transfer matrices of the three sections of the conical transition longitudinal vibration horn, respectively. The elements in the above matrix are as follows:

(12) $$a_{11}^{1}=a_{22}^{1}=\frac{\rho c_{l}S_{1}}{j\tan\left(k_{L}L_{1}\right)}$$

(13) $$a_{21}^{1}=a_{12}^{1}=\frac{\rho c_{l}S_{1}}{j\sin\left(k_{L}L_{1}\right)}$$

(14) $$a_{12}^{2}=a_{21}^{2}=\frac{\rho c_{l}\sqrt{S_{1}S_{2}}}{j\sin\left(k_{L}L_{2}\right)}$$

(15) $$a_{11}^{2}=\frac{\rho c_{l}S_{1}}{j\tan\left(k_{L}L_{2}\right)}-\frac{\rho c_{l}S_{1}\alpha }{jk_{L}}$$

(16) $$a_{22}^{2}=\frac{\rho c_{l}S_{2}}{j\tan\left(k_{L}L_{2}\right)}+\frac{\rho c_{l}S_{2}\alpha N}{jk_{L}}$$

(17) $$a_{11}^{3}=a_{22}^{3}=\frac{\rho c_{l}S_{3}}{j\tan\left(k_{L}L_{3}\right)}$$

(18) $$a_{12}^{3}=a_{21}^{3}=\frac{\rho c_{l}S_{3}}{j\sin\left(k_{L}L_{3}\right)}$$

where *k_l_* = *ω/c_l_*, *k_l_* is the circular wave number; *ω* is the circular frequency; *c_l_* = (*E/ρ*)^1/2^, which is the longitudinal wave propagation velocity in the horn; *ρ* is the density of the material; and *E* is the Young’s modulus.

According to matrix multiplication theory, Equation (11) can be further simplified as follows:

(19) $$\left[\begin{array}{c} F_{3}\\ v_{3} \end{array}\right]=\left[\begin{array}{cc} a_{11}^{*} & a_{12}^{*}\\ a_{21}^{*} & a_{22}^{*} \end{array}\right]\left[\begin{array}{c} F_{1}\\ v_{1} \end{array}\right]$$

where $a_{11}^{*}$, $a_{12}^{*}$, $a_{21}^{*}$ and $a_{22}^{*}$ are the elements obtained by the product of the three impedance transfer matrices of the horn, respectively. According to the boundary conditions of the horn [33,34], the force on the free cross-section at both ends of system is 0, then *F*_1_ = *F*_3_ = 0, $a_{12}^{*}$ = 0, and the longitudinal vibration frequency equation of the conical transition horn is obtained as follows [32]:

(20) $$\tan\left(k_{L}L_{3}\right)=\frac{\left[k_{L}^{2}+\alpha Nk_{L}\tan\left(k_{L}L_{2}\right)\right]\tan\left(k_{L}L_{1}\right)+\left(k_{L}^{2}+\alpha^{2}N\right)\tan\left(k_{L}L_{2}\right)+\left(1-N\right)\alpha k_{L}}{k_{L}^{2}\tan\left(k_{L}L_{1}\right)\tan\left(k_{L}L_{2}\right)+\alpha k_{L}\tan\left(k_{L}L_{2}\right)-1}$$

Further, the amplification factor of longitudinal vibration can be expressed as follows [32]:

(21) $$M_{L}=\left|\frac{N\left\{\cos(k_{L}L_{1})\cos(k_{L}L_{2})-\left[\sin\left(k_{L}L_{1}\right)+\frac{\alpha }{k_{L}}\cos(k_{L}L_{3})\right]\sin\left(k_{L}L_{2}\right)\right\}}{\cos(k_{L}L_{3})}\right|$$

From Equation (20), it can be seen that this equation is a transcendental equation set about the geometric parameters of the horn shape and material characteristic parameters [35]. In general, it is difficult to solve the numerical solution. However, in engineering applications, the material, area coefficient, and working frequency of the composite horn are often pre-set. Therefore, the material characteristic parameters such as sound velocity, density, elastic modulus, and Poisson’s ratio of composite horn are determined. The material parameters of TC4 titanium alloy are shown in Table 2.

Due to the design requirements, it is necessary to match the existing 28 kHz transducer, and the size of the grinding wheel has been determined to be *d*_4_ = 50 mm and *L*_4_ = 15 mm. The known size parameters of the longitudinal-torsional composite ultrasonic horn and grinding wheel are shown in Table 3.

Therefore, there are only three unknowns left in the longitudinal ultrasonic frequency equations: the length of the two cylindrical sections *L*_1_, *L*_3_ and the length of the conical section *L*_2_. In order to obtain the accurate numerical solution of the transcendental equations, the length *L*_1_ of the large cylindrical segment can be determined in advance.

According to traditional horn design theory, the flange is generally set at the zero point of the vibration displacement of the horn, and the position is about 1/4 of the wavelength. According to the material parameters, the full wavelength *λ* of the longitudinal wave can be calculated as:

(22) $$\lambda =\frac{\sqrt{E/\rho }}{f}=176.38\mathrm{mm}$$

It can be seen from Equation (22) that the vibration displacement zero point *X*_0_ is about 44.1 mm. Due to the design and use requirements, the diameter of the flange is *d*_5_ = 70 mm, the length is *L*_5_ = 8 mm, and the vibration displacement zero point *X*_0_ should be in the center of the flange, then the length of the large cylindrical section I can be scheduled as follows:

(23) $$L_{1}=X_{0}+\frac{L_{5}}{2}=48.1\mathrm{mm}$$

Substituting all the above known parameters into Equations (20) and (21), *L*_2_ = 55.114 mm, *L*_3_ = 41.078 mm, and the amplification factor *M_L_* = 1.26 is obtained.

**Figure 3:**
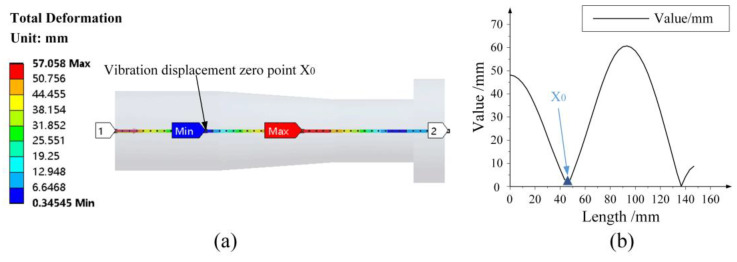
The variation of zero point of vibration displacement after applying tool head; (**a**) The modal analysis result; (**b**) The vibration displacement curve.

According to the predetermined size parameters of the horn, the horn and the grinding wheel are modeled by SolidWorks 2018 software and imported into the finite element analysis software ANSYS 16.0. The path is added in a longitudinal direction, and the modal analysis result is shown in Figure 3a. The vibration displacement zero point has a deviation, which is considered to be the influence of the applied tool head. As shown in Figure 3b, the zero point of vibration displacement changes to 46.667 mm.

**Figure 4:**
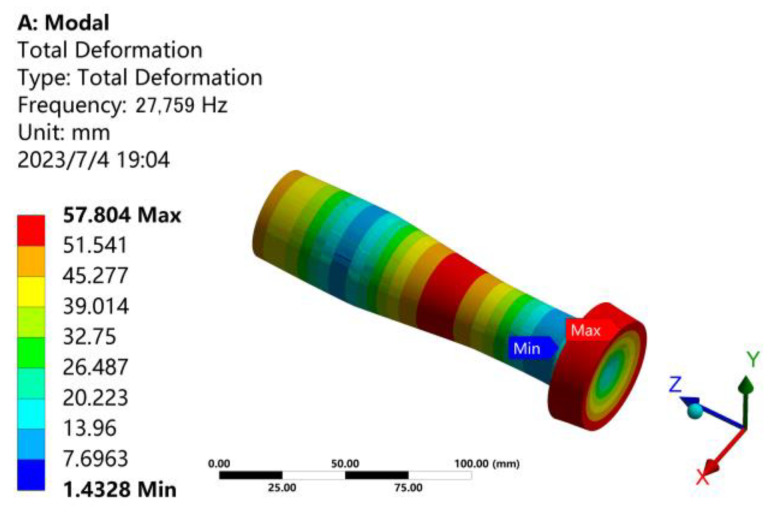
The modal shape of the longitudinal vibration horn with tool head at *f* = 27,759 Hz.

By slightly adjusting the size of each section and performing modal analysis, the vibration mode is continuously optimized, and finally, a better vibration mode is obtained under the size of *L*_1_ = 52 mm, *L*_2_ = 56 mm, and *L*_3_ = 37 mm. The modal analysis result of the longitudinal vibration horn with tool head is shown in Figure 4. The resonance frequency is 27,759 Hz, which is 241 Hz less than the theoretical design frequency of 28,000 Hz, and the error is 0.9%. According to Table 1, the frequency error requirement is 5%. Therefore, the frequency error is within the allowable range and meets the requirements.

The size parameters of each section of the conical transition longitudinal vibration horn with tool head are determined as shown in Table 4:

### 2.3. Design and Optimization of Spiral Groove

**Figure 5:**
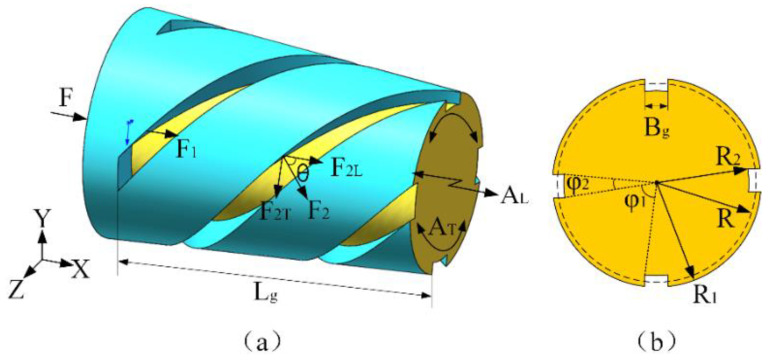
The principle of longitudinal-torsional vibration conversion in conical spiral groove: (**a**) The spiral groove of the conical section; (**b**) The cross-sectional view of the conical section.

In the design process of the longitudinal-torsional ultrasonic vibration horn, the output torsional vibration of the horn is realized through a spiral groove. Some literatures have explained the principle of applying spiral grooves to achieve longitudinal-torsional vibration [21,36]. As shown in Figure 5, the conical transition section can be regarded as composed of a cylindrical section at the bottom of the groove with a radius of *R*_2_ and an outer spiral part. When the longitudinal ultrasonic vibration force *F* is applied to the large cylindrical end of the horn and transmitted to the conical transition section, part of the longitudinal ultrasonic vibration force *F*_1_ continues to be transmitted along the cylindrical section at the bottom of the groove. Due to the angle *θ* of the spiral groove in the outer layer, the longitudinal ultrasonic vibration force is transmitted to the force *F*_2_ perpendicular to the spiral groove surface, and *F*_2_ is further decomposed into the longitudinal force *F*_2L_ and the torsional force *F*_2T_ along the spiral angle *θ* [37]. The longitudinal ultrasonic amplitude *A*_L_ and the torsional ultrasonic amplitude *A*_T_ are output at the end of the small cylinder, so the single longitudinal ultrasonic vibration is converted into longitudinal-torsional ultrasonic vibration through the spiral groove.

According to the torsional vibration theory of ultrasonic vibration, the tangential component *F*_2*T*_ causes the system to produce torsional vibration. Hence. we can use the same method [37] to deduce both the generated torque *M* and longitudinal-torsional mechanical conversion coefficient *n_T_* as follows:

(24) $$M=\int_{R_{2}}^{R_{1}}R\cdot \frac{90F_{2}\sin\theta }{{\pi R^{2}\varphi }_{1}+{\pi R_{2}^{2}\varphi }_{2}}\cdot \frac{\pi R\varphi_{1}}{45}dR=2F_{2}\sin\theta \left[\left(R_{1}-R_{2}\right)-R_{2}\sqrt{\frac{\varphi_{2}}{\varphi_{1}}}\arctan\left(\frac{R_{1}}{R_{2}}\cdot \sqrt{\frac{\varphi_{1}}{\varphi_{2}}}\right)-\arctan\sqrt{\frac{\varphi_{1}}{\varphi_{2}}}\right]$$

(25) $$n_{T}=\frac{M}{F}=\frac{2F_{2}\sin\theta \left[\left(R_{1}-R_{2}\right)-R_{2}\sqrt{\frac{\varphi_{2}}{\varphi_{1}}}\arctan\left(\frac{R_{1}}{R_{2}}\cdot \sqrt{\frac{\varphi_{1}}{\varphi_{2}}}\right)-\arctan\sqrt{\frac{\varphi_{1}}{\varphi_{2}}}\right]}{F}$$

It can be seen from Equation (25) that the longitudinal-torsional mechanical conversion coefficient *n_T_* is related to *R*_1_, *R*_2_, *φ*_1_, *φ*_2_, and *θ*. That is, it is related to the groove depth, the number of the spiral grooves, the groove width, and the spiral angle. As shown in Figure 5, the width of the spiral groove is *B_g_*, the length is *L_g_*, the number is *N* (generally *N* = 4), and the spiral angle is *θ*. Since the conical section has a tilt angle, the depth of the spiral groove is not a fixed value, so the radius *R*_2_ of the spiral groove bottom cylinder is used to characterize the depth of the spiral groove. The larger *R*_2_ is, the smaller the depth of spiral groove; the smaller *R*_2_ is, the greater the depth of the spiral groove.

When selecting the parameters of the spiral groove, the influence of the spiral groove on the design frequency *f* and the torsional-longitudinal ratio *i* (*i* = *A_T_*/*A_L_*) is the main target. Relevant studies have shown that, as the spiral angle *θ* of the spiral groove increases from small to large, *i* increases first and then decreases, and the torsional-longitudinal ratio *i* reaches the maximum when the spiral angle *θ* = 45°. As the length of the spiral groove *L_g_* increases, the torsional-longitudinal ratio *i* increases [23,38]. Therefore, the number of the spiral grooves is *N* = 4, the spiral angle *θ* = 45°, and the length of the spiral groove *L_g_* is as long as possible. The length of the conical section is known to be *L*_2_ = 56 mm. Considering the actual machining operation and the presence of the tool radius, a safety clearance of 6 mm is reserved and the length of the spiral groove is *L_g_* = 50 mm. From the relevant literature [21,23], it is found that the most commonly selected range of spiral groove width is 4–8 mm. And, the depth of the spiral groove also has an effect on the frequency and torsional-longitudinal ratio [21]. The maximum value of *R_2_* is selected to be equal to the radius of the small cylindrical section of 15 mm, and in order to make the conical transition section III have sufficient strength, the minimum value of *R_2_* is selected to be 12 mm. Hence, the value of *R*_2_ is selected in the range of 12–15 mm. The parameter optimization of the width of the spiral groove, *B_g_*, and the radius of bottom cylinder of spiral groove, *R*_2_, is carried out using single factor simulation analysis approach [39]. Parameters to be optimized are shown in Table 5.

**Figure 6:**
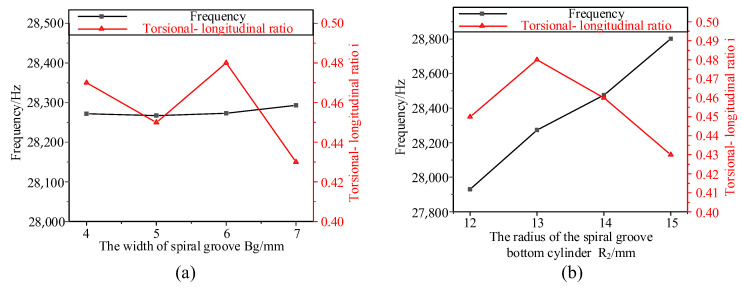
The effect of different *B_g_* and *R*_2_ on frequency *f* and torsional-longitudinal ratio *i*: (**a**) *B_g_*; (**b**) *R*_2_.

The results of the ANSYS analysis are shown in Figure 6. In Figure 6a, with the increase in *B_g_*, the frequency *f* changes little; And, *i* decreases first and then increases and then decreases again and reaches the maximum at *B_g_* = 6 mm, so the spiral groove width *B_g_* is determined to be 6 mm. Then, the effects of different *R*_2_ on frequency *f* and torsional-longitudinal ratio *i* are analyzed. As shown in Figure 6b, with the increase in *R*_2_, the frequency *f* also increases, and the torsion ratio *i* increases first and then decreases, reaching the maximum when *R*_2_ = 13 mm. Therefore, the parameters of the spiral groove are all determined, as shown in Table 6.

## 3. Finite Element Analysis of Longitudinal-Torsional Composite Ultrasonic Horn with Tool Head

### 3.1. Modal Analysis

**Figure 7:**
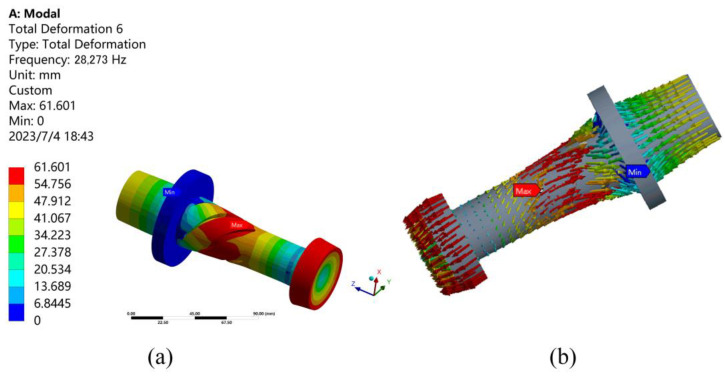
The modal analysis results: (**a**) The modal analysis results at *f* = 28,273 Hz; (**b**) The vibration vector arrows in the longitudinal and torsional directions of the horn.

All parameters of the conical transition longitudinal-torsional composite horn have been determined. Because the flange plays a fixed role, SolidWorks 2018 software is used to establish the model together with the horn, then imported into the finite element analysis software ANSYS 16.0 for modal analysis. In the modal analysis setting, fixed constraints are applied to the flange in order to fit the actual situation, and the modes are searched for a total of 30 orders. As shown in Figure 7, the best vibration mode appears in the 19-order modal analysis.

As shown in Figure 7a, the resonance frequency is 28,273 Hz, with a difference of 273 Hz from the theoretical design frequency of 28,000 Hz, the error is only 0.98%, which is less than 5% and meets the design requirements. And, as shown in Figure 7b, the vibration vector arrows can be clearly seen in the longitudinal and torsional directions. The grinding wheel has good longitudinal-torsional compound vibration characteristics.

### 3.2. Transient Dynamic Analysis

**Figure 8:**
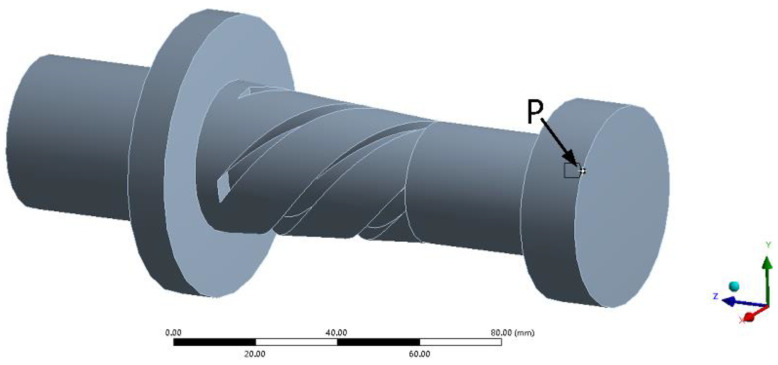
The particle *P* selected on the outer circle of the grinding wheel.

**Figure 9:**
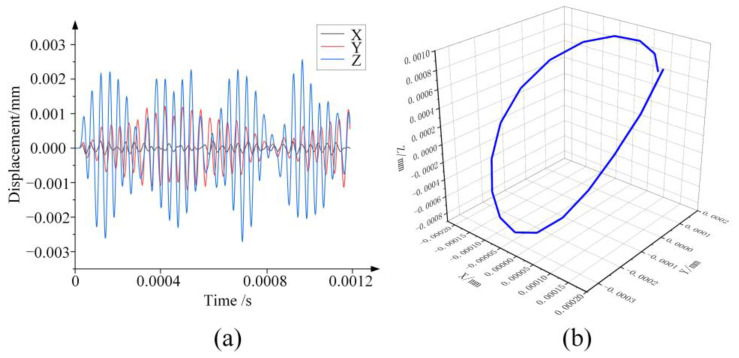
The transient dynamic analysis results: (**a**) The vibration displacement of particle *P*; (**b**) The vibration displacement trajectory of particle *P* in space during one cycle.

As shown in Figure 8, in order to analyze the vibration characteristics of the outer circle of the grinding wheel, a point *P* on the outer circle of the grinding wheel is selected for transient dynamic analysis. The longitudinal displacement excitation u(x)=Asin(2πft) (where *A* = 1 μm, *f* = 28,273 Hz) is applied to the large end of the horn. And, the vibrational displacement of particle *P* on the grinding wheel in the X, Y and Z directions is the output. The transient dynamics analysis takes 30 cycles, and each cycle is divided into 20 time periods. The analysis results are shown in Figure 9.

From the analysis results, the vibration displacement in the Z-axis direction is the longitudinal vibration amplitude *A_L_* of particle *P* and the vibration displacement in the Y-axis direction is the torsional vibration amplitude *A_T_* of particle *P*. From Figure 9a, the torsional-longitudinal ratio *i* = *A_T_*/*A_L_* = 0.48. As shown in Figure 9b, the spatial vibration displacement trajectory of particle *P* in one period is an approximate elliptic curve.

## 4. Vibration Performance Test

### 4.1. Impedance Analysis

**Figure 10:**
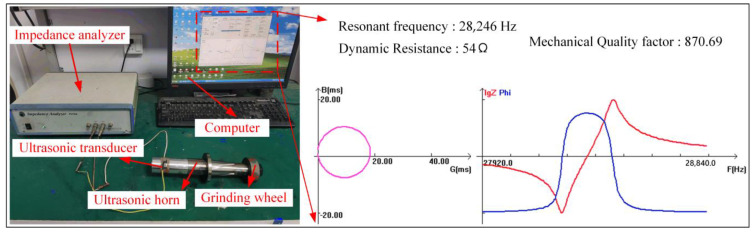
The impedance analysis test site and test results.

In order to verify the results of the finite element simulation, the horn was processed according to the above parameters. The impedance characteristic of the horn was measured by the impedance analyzer PV70 (Beijing Band Era Co., Ltd., Beijing, China) shown in Figure 10. It can be seen that the resonant frequency is 28,246 Hz, and the frequency error is 0.88% compared with the theoretical design, which is less than the allowable error range of 5%. There is no parasitic circle in the admittance circle, and the roundness is good. The conductance curve is normal, and the dynamic resistance is only 54 Ω, indicating that the heat loss of the system is small. The mechanical quality factor of the vibration system is 870, which indicates that the electro-acoustic conversion efficiency of the ultrasonic vibrator of the system is high. According to Table 1, the above characteristics are within the scope of design requirements, indicating that the size and structure of the designed horn are reasonable.

### 4.2. Amplitude Test

**Figure 11:**
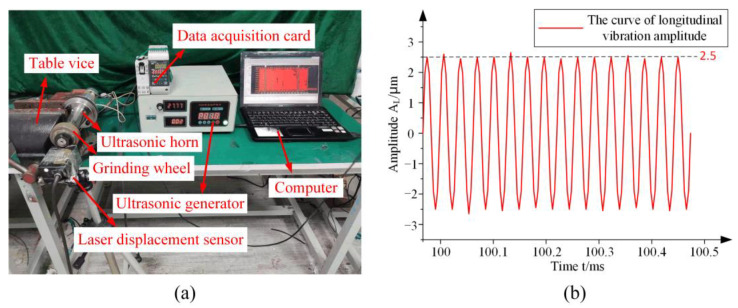
The longitudinal ultrasonic amplitude test: (**a**) The longitudinal ultrasonic amplitude test site; (**b**) The longitudinal amplitude test results.

As shown in Figure 11a, the amplitude measurement device consisted of the laser displacement sensor (LK-G10, KEYENCE, Osaka, Japan), an ultrasonic generator, and a computer. The laser displacement sensor was used to focus a laser beam on the output end-face of the grinding wheel. In order to simulate the fixed constraints of the flange during simulation and actual installation, a table vice was used to clamp the flange for more accurate amplitude measurements. After the output of the ultrasonic generator is stable, the amplitude test was carried out.

**Figure 12:**
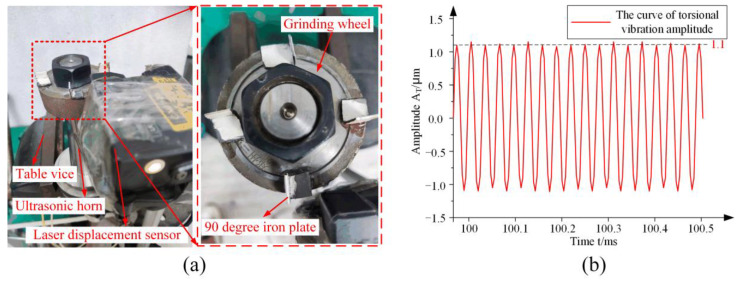
The torsional amplitude test: (**a**) The torsional amplitude test site; (**b**) The torsional amplitude test results.

The torsional ultrasonic amplitude test method was similar to the longitudinal ultrasonic amplitude test method. However, the difference was that because the grinding wheel was circular, the laser displacement sensor could not directly measure torsional ultrasonic amplitude. Therefore, as shown in Figure 12a, four 90° small iron sheets were uniformly adhered to the circumferential direction of the grinding wheel end-face by strong adhesive. After the small iron piece was tightly bonded to the grinding wheel, the laser displacement sensor was used to irradiate the laser as close as possible at the bonding place between the small iron piece and the outer circle of the grinding wheel. Each 90° small iron sheet was measured separately.

The measurement results of the longitudinal ultrasonic amplitude and torsional ultrasonic amplitude are shown in Figure 11b and Figure 12b, respectively. The measurement results show that the value of the longitudinal amplitude is 2.5 μm, the value of the torsional amplitude is 1.1 μm, and the torsional-longitudinal ratio *i* = 0.44.

## 5. Longitudinal-Torsional Ultrasonic Vibration Internal Grinding Experiment of GCr15 Steel

### 5.1. Experimental Conditions

**Figure 13:**
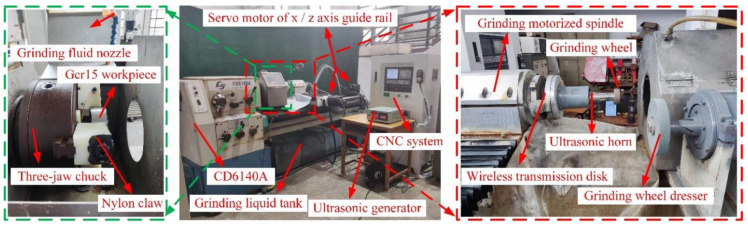
The longitudinal-torsional ultrasonic vibration grinding experiment.

As shown in Figure 13, the longitudinal-torsional ultrasonic vibration internal grinding test of GCr15 steel was carried out on a machine tool modified from a CD6140A lathe. The GSK980TDC numerical control system control cabinet, a grinding wheel dresser, an X/Z axis grinding processing platform, and the longitudinal-torsional ultrasonic horn were auxiliary configured. During the machining process, the horn and the grinding wheel were driven by the grinding motorized spindle to rotate at a high speed, and the precision chuck and the insulating jaw clamped the workpiece and drove it to rotate. The X-axis servo motor drove the motion platform and the grinding motorized spindle through the precision ball screw and the linear guide and realized the radial feed motion of the grinding wheel. The Z-axis servo motor drove the workbench and motorized spindle to move along the longitudinal direction through the precision ball screw, so as to realize the rapid positioning of the grinding wheel. The machining shield was used to prevent the grinding fluid from splashing, and the grinding fluid was collected into the grinding fluid tank through the reflux pipeline. The grinding dresser was used to perform surface modification after the grinding wheel was worn during the machining process to ensure the equal height of the abrasive grinds on the grinding wheel surface.

### 5.2. Experimental Scheme

**Figure 14:**
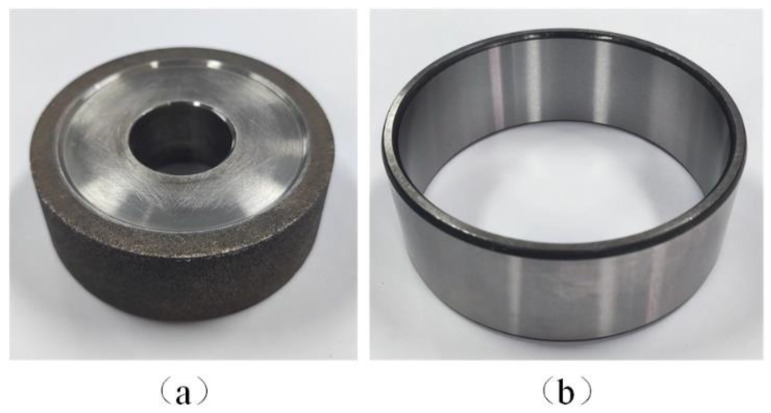
The grinding wheel and GCr15 steel workpiece: (**a**) Grinding wheel; (**b**) GCr15 steel workpiece.

The conical transition longitudinal-torsional composite ultrasonic horn designed in this paper was used in the experiment. The tool was a titanium alloy matrix and bronze bond CBN grinding wheel, as shown in Figure 14a. The grinding wheel had a diameter of 50 mm, a width of 15 mm and was 100# in grit size. The workpiece was a GCr15 steel bearing ring, as shown in Figure 14b, with an inner diameter of 100 mm, an outer diameter of 110 mm, and a width of 40 mm. The material properties of the workpiece are shown in Table 7.

The experiment used longitudinal feed internal grinding. In order to obtain better surface quality, the down-grinding method for the grinding wheel and workpiece was adopted. In order to test the processing performance of the horn, the ultrasonic amplitude was used as single variable to carry out single factor experiment. The experimental processing parameters are shown in Table 8.

After the experiment was completed, a white-light interference microscope (TALYSURF.CCI.6000 by Taylor Hobson Ltd., Leicester, UK) was used to observe the grinding surface morphology of the workpiece and measure the surface roughness value. The effects of different ultrasonic amplitudes on the grinding surface morphology and surface roughness were investigated to verify the vibration machining performance of the horn.

### 5.3. Experimental Results and Discussion

**Figure 15:**
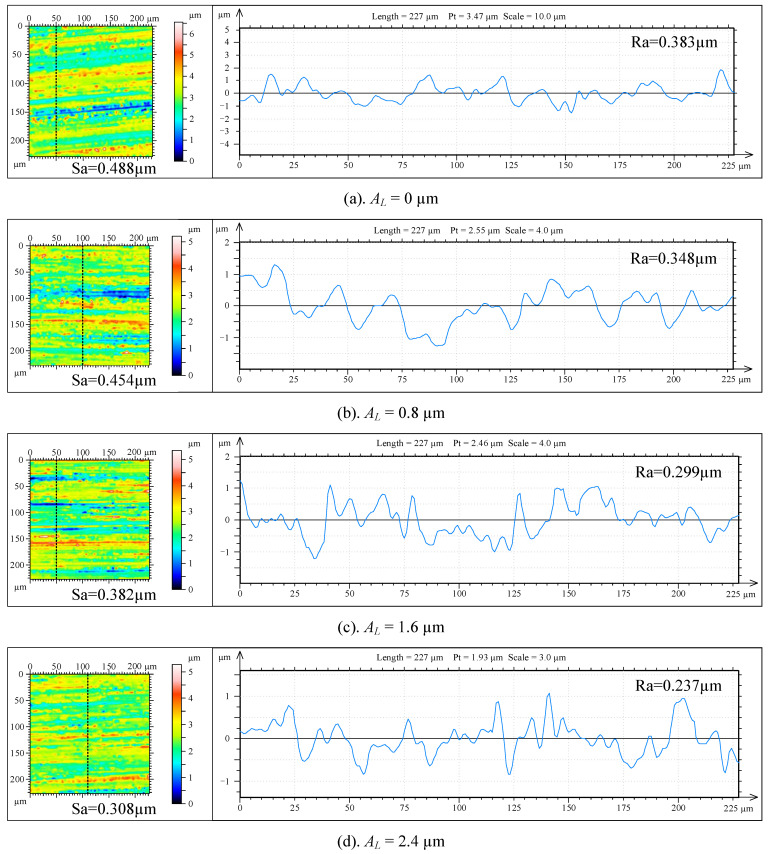
The micromorphology of the workpiece grinding surface under different ultrasonic amplitudes.

As shown in Figure 15, the grinding surface micromorphology of the workpiece was observed with a white light interferometer under different longitudinal-torsional ultrasonic amplitudes, and the surface roughness was measured.

It can be seen from Figure 15a that when *A_L_* = 0 μm, the height difference of the micro-surface of the workpiece is 6.5 μm, and there are obvious gullies and bulges. The roughness curve fluctuates greatly, and the mean surface roughness is large. As shown in Figure 15b–d, the surface micromorphology gradually becomes flatter, with gullies and bulges of gradually decreasing amplitude, while increasing the longitudinal ultrasonic amplitude to 0.8, 1.6 and 2.4 mm. The fluctuation of the surface roughness curve is gradually gentle. The arithmetic mean of surface profile height decreases gradually.

**Figure 16:**
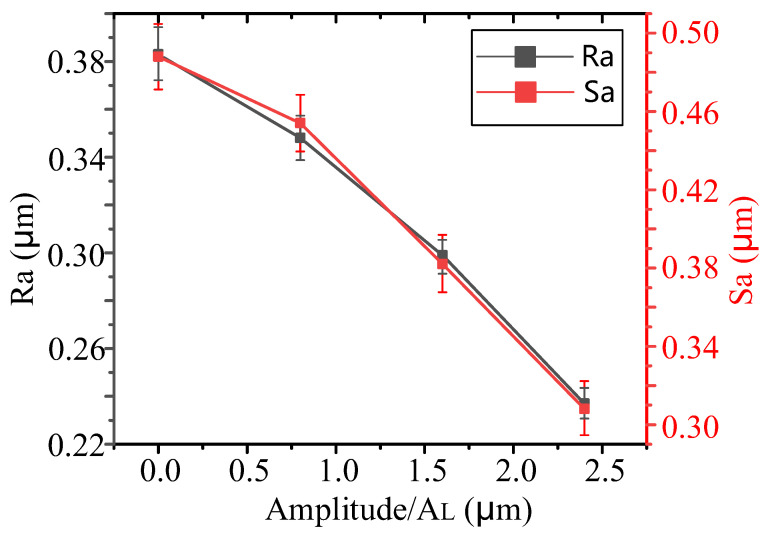
The effects of different amplitudes on grinding surface roughness.

As shown in Figure 16, as the longitudinal ultrasonic amplitude *A_L_* increases from 0 μm to 2.4 μm, the surface roughness *Ra* decreases from 0.383 μm to 0.237 μm, with a decrease of 38.1%, and *Sa* decreases from 0.488 μm to 0.308 μm, with a decrease of 36.9%.

In summary, the grinding surface roughness after applying longitudinal-torsional ultrasonic vibration is significantly less than that of ordinary grinding, and the surface quality is greatly improved. This is because the application of longitudinal-torsional ultrasonic vibration changes the trajectory of abrasive grits and increases the contact arc length between a single abrasive grit and the workpiece [40]. Moreover, due to the mutual interference of different abrasive grits on the surface of the workpiece, the residual height of the surface material of the workpiece is further removed, so that the surface roughness is greatly reduced. With the increase in ultrasonic amplitude in a certain range, the contact arc length between the abrasive particle and the workpiece increases, the vibration displacement of the abrasive particle increases, the average chip thickness decreases, the coincidence rate of the trajectory between the abrasive particles increases, and the surface consistency is improved. The surface quality is gradually improved. These results are similar to those previously reported in the literature [2,41].

## 6. Conclusions

In this paper, the conical transition longitudinal-torsional ultrasonic horn for grinding was designed and optimized by combining transfer matrix theory and finite element simulation. The vibration performance test and the grinding experiment were carried out. Finally, the main conclusions of this study can be summarized as follows: 

(1) Based on the transfer matrix theory and ANSYS finite element simulation analysis, the longitudinal-torsional composite ultrasonic conical transition horn was designed and optimized. The modal analysis and transient dynamic analysis were carried out. And, the analysis results showed that the designed horn had better vibration characteristics.

(2) The impedance analysis results showed that the resonant frequency *f* was 28,246 Hz and the error was 0.88% compared to the theoretical design frequency. The amplitude test results showed that the maximum amplitude of the grinding wheel (*d*_4_ = 50 mm, *L*_4_ = 15 mm) was 2.5 μm of *A_L_* and 1.1 μm of *A_T_*. The torsional-longitudinal ratio *i* was 0.44.

(3) The longitudinal-torsional ultrasonic vibration internal grinding experiment of GCr15 results showed that the surface roughness of the workpiece decreased with the increase in amplitude. Compared with ordinary grinding, the surface roughness of the longitudinal-torsional ultrasonic grinding workpiece was reduced by a maximum of 38.1%. The experiment verifies the correctness of the theory and simulation and proves the great processing performance of the horn.

## Figures and Tables

**Table 1:** The requirements for vibration and impedance characteristics of the horn.

Characteristic	Frequency (Hz)	Dynamic Resistance (Ω)	Mechanical Quality Factor
Value	28,000 Hz ± 5%	5–100	500–1000

**Table 2:** The material parameters of TC4 titanium alloy.

Material	Density (kg/m^−3^)	Sound Velocity (m/s)	Poisson Ratio	Elastic Modulus (Gpa)
TC4 titanium alloy	4510	6100	0.34	110

**Table 3:** Determined horn size parameters.

Parameter	*d*_1_	*d*_3_	*d*_4_	*L*_4_
mm	38	30	50	15

**Table 4:** The dimension parameters of the conical transition longitudinal vibration horn with tool head.

Parameter	*d*_1_	*d*_3_	*d*_4_	*L*_1_	*L*_2_	*L*_3_	*L*_4_
mm	38	30	50	52	56	37	15

**Table 5:** Parameters to be optimized of spiral grooves.

Parameter	Value			
*R*_2_/mm	12	13	14	15
*B_g_*/mm	4	5	6	7

**Table 6:** The parameters of spiral groove.

Parameter	Value	Unit
*B_g_*	6	mm
*L_g_*	50	mm
*N*	4	/
*θ*	45	°
*R*_2_	13	mm

**Table 7:** The material properties of GCr15 steel.

Property	Value	Unit
Young’s modulus	208	Gpa
Hardness	60–65	HRC
Density	7.81	g/cm^3^
Poisson ratio	0.3	/

**Table 8:** The parameters of grinding experiments.

Parameter	Value	Unit
Longitudinal ultrasound amplitude *A_L_*	0, 0.8, 1.6, 2.4	μm
Torsional-longitudinal ratio *i*	0.44	/
Ultrasonic frequency *f*	28	kHz
Grinding wheel speed *n_s_*	6000	r/min
Workpiece speed *n_w_*	35	r/min
Grinding depth *a_p_*	10	μm
Longitudinal feed rate *V_f_*	50	mm/min

## Data Availability

Not applicable.
